# Leucine auxotrophic marker for genetic manipulation of *Mycobacterium abscessus*

**DOI:** 10.1128/aem.00712-26

**Published:** 2026-07-16

**Authors:** Tizian Griesser, Petra Selchow, Peter Sander

**Affiliations:** 1 Institute of Medical Microbiology, University of Zurich, Zurich, Switzerland; 2 National Reference Laboratory for Mycobacteria, University of Zurich 27217, Zurich, Switzerland; Indiana University Bloomington, Bloomington, Indiana, USA

**Keywords:** *Mycobacterium abscessus*, auxotrophic marker, genetic tool, leucine auxotroph, aminoglycoside resistance

## Abstract

*Mycobacterium abscessus* is a clinically important multidrug-resistant pathogen for which genetic manipulation remains challenging. Here, we developed a second-generation genetic toolbox based on leucine auxotrophy that enables antibiotic-free positive selection. A Δ*leuB* mutant, lacking the gene encoding isopropyl malate dehydrogenase in the leucine biosynthesis pathway, was generated by targeted gene deletion. This mutant requires exogenous leucine for growth and can be complemented by plasmid-borne *leuB*, establishing a robust auxotrophy-based selection system. To support genetic manipulation, we constructed a suite of second-generation vectors, including a multicopy replicative vector (pRep-amp-*leuB*), a single-copy integrative vector (pInt-amp-*leuB*), and a suicide vector (pSuc-amp-*leuB*) for allelic replacement. These vectors enable gene overexpression, complementation, and targeted gene deletion, respectively, without reliance on aminoglycoside resistance markers. Using this system, we demonstrate efficient transformation and functional complementation of the Δ*leuB* mutant, achieving high transformation efficiencies and near-zero background growth under leucine selection. In contrast to antibiotic-based systems, this approach eliminates nonspecific background and avoids activation of stress response pathways, such as the *whiB7* regulon. Overall, this auxotrophy-based toolbox provides a versatile platform for precise genetic manipulation in *M. abscessus*, improving selection stringency and enabling antibiotic-free functional genomics approaches, as demonstrated by deleting *aac(2*′), *eis2,* and a 19-kb fragment of the *gpl* locus.

**IMPORTANCE:**

The development of a leucine auxotroph-based genetic system for *Mycobacterium abscessus* addresses critical challenges in mycobacterial genetics. By avoiding established antibiotic resistance markers, this approach reduces selective pressure for antibiotic-resistant transformants, supports antibiotic stewardship, and minimizes costly disposal from laboratory waste. It also avoids unintended activation of *whiB7*, a master regulator of approximately 100 genes, particularly those involved in antibiotic stress responses, thereby improving the accuracy of phenotypic drug susceptibility testing. The versatile genetic toolbox developed here, including novel replicative, integrative, and suicide plasmids, provides precise control over functional studies, overexpression, complementation, and gene deletion. It significantly reduces dependency on antibiotics for genetic manipulation, aligning with the goals of sustainable research and offering new opportunities for studying this clinically significant pathogen. This approach represents a critical advance in microbial genetics, enhancing our capacity to explore the molecular basis of pathogenesis and drug resistance in *M. abscessus*.

## INTRODUCTION

*Mycobacterium abscessus* is a rapidly growing, multidrug-resistant, non-tuberculous mycobacterium (NTM) that is associated with severe pulmonary infections, particularly in individuals with underlying lung conditions such as cystic fibrosis (1, 2) and chronic obstructive pulmonary disease (COPD) (3). This opportunistic pathogen belongs to risk group 2 (4) due to its ability to cause nosocomial infections and its intrinsic resistance to multiple antibiotics (5). The low permeability of the waxy and hydrophobic mycobacterial cell wall, drug export systems, and drug- and drug target-modifying enzymes contribute to its resistance to tuberculosis first-line drugs rifampicin and isoniazid, most β-lactams, tetracyclines, aminoglycosides, and macrolides (6). This has led to the recognition of *M. abscessus* as a major cause of concern in the field of medicine, often referred to as the antibiotic and clinical nightmare (7, 8).

The treatment of *M. abscessus* infections is a highly challenging endeavor, often necessitating prolonged and complex antibiotic regimens with limited success rates (9). *M. abscessus* can be further divided into three subspecies: *M. abscessus subsp. abscessus, M. abscessus subsp. bolletti*, and *M. abscessus subsp. massiliense*. Subspecies identification is clinically relevant, as *M. abscessus subsp. massiliense* harbors a non-functional *erm(41*) gene, resulting in susceptibility to macrolides and consequently positively influencing treatment outcome (10), while the other two subspecies possess a functional *erm(41)*. The *erm(41*) encodes an erythromycin ribosomal methylase that methylates adenine 2058 in the 23S rRNA, thereby preventing macrolides from binding to their ribosomal target site (11). Expression of *erm(41*) is inducible and activated upon exposure to macrolides (11) in a *whiB7*-dependent manner. Currently, an established standard recommendation for treating *M. abscessus* infection does not exist (12). The American Thoracic Society has recommended clarithromycin, amikacin, and cefoxitin for initial therapy (up to 3 months), followed by the use of tigecycline, clofazimine, and linezolid for the continuous phase (12 months and more after culture conversion) (13). Clinically acquired resistance to amikacin and macrolides is frequently attributable to mutations in the drug target sites *rrs* and *rrl*, encoding 16S rRNA and 23S rRNA, respectively (6). In light of mounting concerns regarding antimicrobial use and resistance, the stewardship of antibiotics has emerged as a pivotal facet of microbiological research and clinical management of *M. abscessus (14*). The regulation of heat-stable antibiotics, along with the legally mandated systems for their collection and disposal, is a critical part, but also costly, of the global effort to minimize environmental contamination and antimicrobial resistance.

Genetic manipulation of *M. abscessus* and characterization of isogenic mutants has been essential for understanding its pathogenicity, resistance mechanisms, and the therapeutic outcome (15). The present genetic toolkit for this microbe principally relies on antibiotic selection markers for plasmid maintenance and chromosomal integration (16, 17). These include resistance genes for animal-used and laboratory antibiotics, such as apramycin, kanamycin, hygromycin, and zeocin, which enable the positive selection for transformed bacteria (18–20). In the context of generating deletion mutants, the KatG-dependent isoniazid counterselection or sucrose-dependent (*sacB*) method is employed (16, 21). The continued use or environmental presence of certain antibiotics can maintain or promote resistance not only to those drugs but also to other clinically important antibiotics, due to genetic linkage of resistance genes (co-selection) or mechanisms that act across antibiotic classes (cross-resistance). Despite its widespread use, antibiotic selection in genetic studies of *M. abscessus* still is challenging and requires additional screening due to high background levels from spontaneous resistance. The use of antibiotics increases the environmental burden and is associated with increasing costs for disposal of antibiotic waste material.

The *whiB7* regulon in *M. abscessus* represents a key regulatory system involved in the bacterial response to antibiotic stress (22, 23). It encodes a transcriptional regulator that is induced by a broad range of antibiotics, including aminoglycosides and macrolides, as well as additional stress conditions. Upon activation, WhiB7 upregulates the expression of numerous resistance-associated genes, including efflux systems, ribosomal protection factors, aminoglycoside-modifying enzymes, and the *erm(41*) macrolide resistance determinant (24). Through this coordinated response, the *whiB7* regulon contributes to intrinsic multidrug resistance and adaptive tolerance mechanisms in *M. abscessus*. While induction of this regulon does not inherently invalidate antibiotic susceptibility testing, prior exposure to inducing agents may influence bacterial physiology and gene expression states. As a result, antibiotic-based selection strategies may introduce context-dependent effects in downstream phenotypic assays, particularly in studies of antimicrobial response and stress adaptation.

Intrinsic resistance to aminoglycosides in *M. abscessus* is primarily mediated by aminoglycoside-modifying enzymes, many of which exhibit overlapping substrate specificities (25). The *aac(2′),* in particular, encodes an aminoglycoside acetyltransferase, a key enzyme that modifies and inactivates aminoglycosides, such as kanamycin B and tobramycin, thereby reducing their efficacy (25). The *eis2* encodes an enhanced intracellular survival protein, belonging to the Eis protein family, which was shown in *Mycobacterium tuberculosis* to be involved in resistance toward amikacin and kanamycin A (26). This broad-spectrum N-acetyl transferase modifies multiple aminoglycosides, including the clinically important amikacin, thereby contributing to resistance and affecting treatment outcomes in pre-clinical models of *M. abscessus* infection (27). The presence of these resistance determinants and their regulators complicates antibiotic-based selection strategies and underscores the need for alternative selection methods.

Furthermore, the management of waste materials containing antibiotics is a critical concern in research and is subject to hazardous waste legislation. In Europe, this includes the EU Waste Framework Directive (2008/98/EC), as well as chemical safety regulations such as REACH. In the United States, it falls under the Resource Conservation and Recovery Act (RCRA). A significant proportion of antibiotics used for laboratory selection are heat-stable, meaning they are not inactivated by autoclaving. This necessitates specialized disposal procedures, thereby imposing additional logistical and financial burdens on research activities. Consequently, there is a considerable need to simplify waste management and reduce antibiotic consumption to achieve more sustainable research practices.

To address all the challenges, we propose a new leucine auxotrophic marker system for *M. abscessus* genetic manipulation. While leucine auxotrophy has frequently been generated by targeting the *leuCD* genes, we chose to focus on *leuB*. The *leuB* gene encodes a single, non-redundant enzyme in the leucine biosynthesis pathway, allowing the generation of a stable auxotrophic phenotype through disruption of a single genetic locus. This strategy minimizes genetic perturbation and facilitates precise genetic complementation compared with multi-gene deletions. This alternative selection strategy aims to minimize the use of antibiotics, reduce background growth issues, and streamline waste management. By leveraging auxotroph-based selection, a reliable and environmentally sustainable genetic system for *M. abscessus* research can be established. This study details the construction and validation of a leucine auxotrophic *M. abscessus* mutant of the widely used *M. abscessus* ATCC 19977 strain and application of leucine prototrophy (Leu^+^) as a selectable marker for genetic engineering.

## RESULTS

### Selection of a metabolic pathway for auxotrophy in *Mycobacterium abscessus*

**Fig 1:**
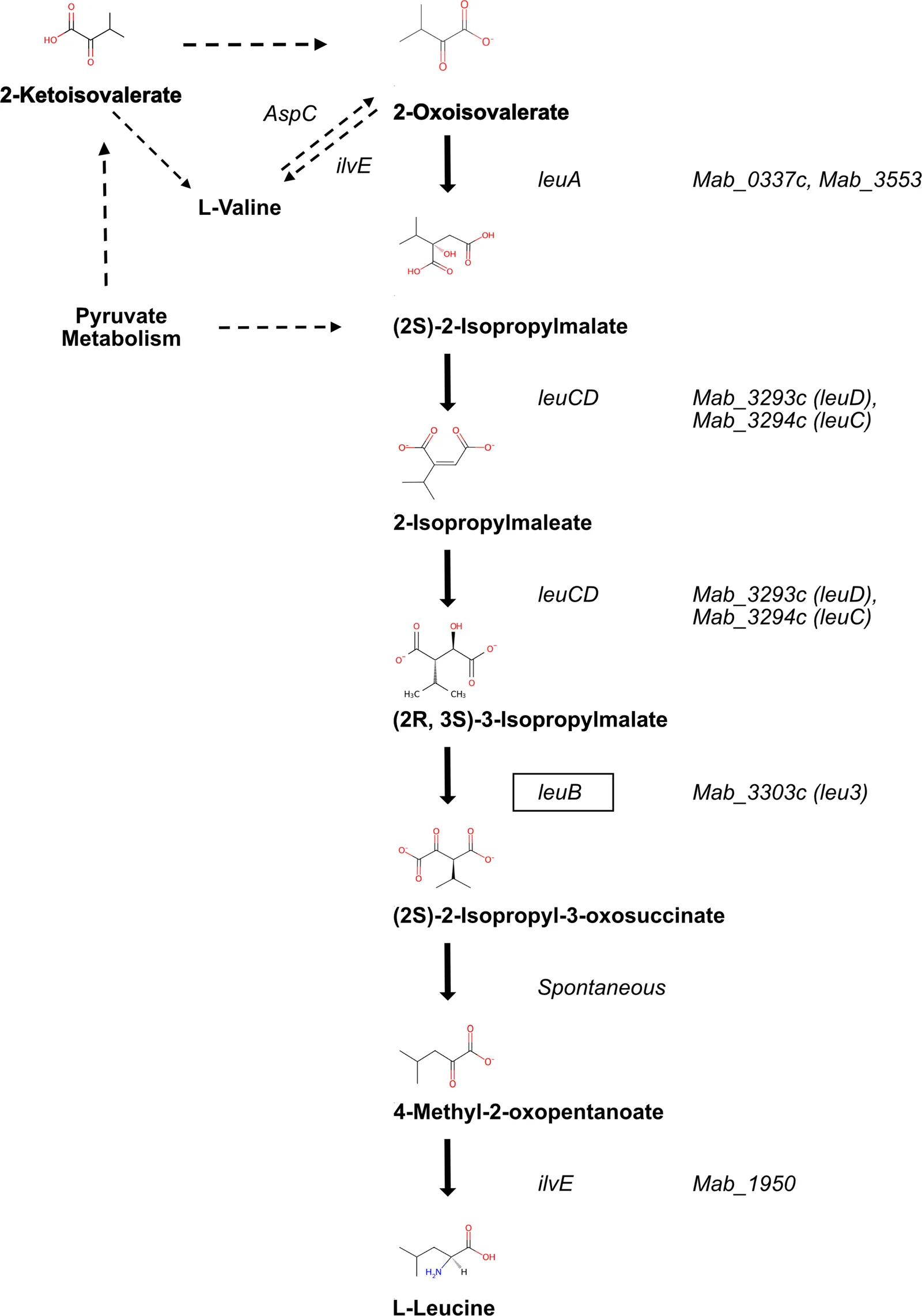
Leucine biosynthesis pathway and corresponding genes of *M. abscessus*. *leuA*, *leuCD*, and *leuB* are genes involved in the biosynthesis of L-leucine and are not shared by the synthetic pathway of the other branched amino acids like valine or isoleucine. The leucine pathway is provided with 2-oxoisovalerate mainly from the valine biosynthetic pathway, the terminal intermediate (with pyruvate as the starting molecule), or from degradation of valine itself. *leuB* converts 3-isopropylmaleate to 2-ketoisocaproate by first alcohol oxidation followed by spontaneous decarboxylation and tautomerization.

The objective of this study is to engineer an auxotrophic *M. abscessus* ATCC-derived strain and to develop the appropriate genetic toolbox for manipulating this auxotroph. This new setup will reduce the amount of antibiotics used in cloning. An auxotrophic strain is a mutant organism that has lost the ability to synthesize an essential metabolite, such as an amino acid, nucleotide, or vitamin, required for growth. Consequently, the growth of an auxotroph depends on an external supply of the missing compound in the growth medium. We focused on identifying a pathway amenable to complementation through external supplementation of its terminal metabolite. Numerous documented auxotrophic *M. tuberculosis* strains deficient in synthesizing amino acids, particularly in the synthesis of branched amino acids, have been engineered (28). These have been successfully attenuated to yield a viable strain. The enzymes involved in the synthesis of branched amino acids are currently being discussed and evaluated as valuable drug targets (28). Corresponding *M. abscessus* mutants have only recently been described (29). In light of these findings, we have opted to target the synthesis pathway of one of the three branched amino acids, L-leucine (Fig. 1). A total of eight enzymes have been identified for branched amino acid biosynthesis, of which four are shared, and three are exclusively involved in L-leucine biosynthesis (LeuA, LeuB, and LeuCD). The *leuA–leuD* genes are widely conserved across the bacterial phyla, and the enzymes they encode exhibit significant sequence and structural similarities, reflecting the critical role of leucine biosynthesis in bacterial metabolism (30, 31). Several groups have succeeded in creating leucine auxotrophic bacteria by targeting *leuCD* (32). In contrast, our approach was to target *leuB*, which encodes isopropyl malate dehydrogenase, the enzyme that converts the product of LeuCD, 3-isopropylmalate, to 2-ketoisocaproate.

### Generation of *M. abscessus* MAB_3303c (*leuB*) deletion mutant

**Fig 2:**
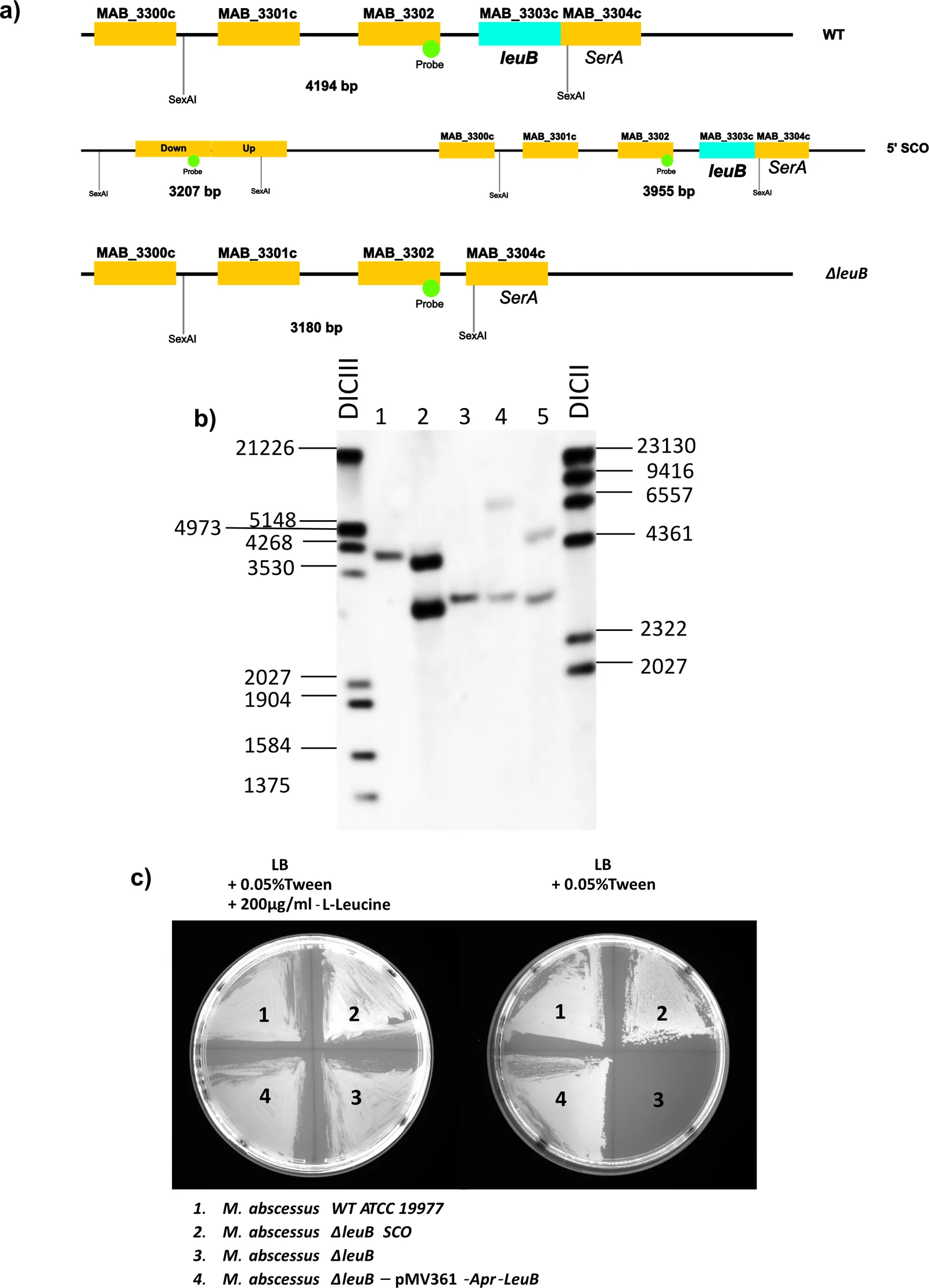
Genotypic and phenotypic characterization of *M. abscessus* Δ*leuB*. (**a**) Schematic genomic events. (**b**) Southern blot analysis of *leuB* deletion mutant. Genomic DNA of *M. abscessus* parental strain (WT, lane 1), after first homologous recombination (SCO, lane 2), after isoniazid counterselection (Δ*leuB*, lane 3), and complementation with either pMV361-Apr-leuB (lane 4) or pInt-leuB (lane 5) were digested with SexAI. Probe was designed to bind in the flanking region of leuB in the ORF of MAB_3302 (green dot). Expected fragments were for WT 4,194 bp (lane 1), 5′ SCO 4,194 and 2,968 bp (lane 2), Δ*leuB* 3,180 bp (lane 3), for pMV361-Apr-leuB complementation 3,180 and 8,522 bp (lane 4), and for pInt-Amp-leuB complementation 3,180 and 5,348 bp (lane 5), respectively. All expected fragments were detected. (**c**) Phenotypic characterization of Δ*leuB* mutant (leu^−^) on agar plates.

The generation of a leucine auxotrophic *M. abscessus* strain necessitates the deletion of the essential *leuB* by conventional techniques, for example, using antibiotic selection. The *M. abscessus* Δ*leuB* mutant was constructed by transformation of *M. abscessus* ATCC 19977 with *leuB* targeting plasmid pKH-Apr-DsRed2-KatG-MAB_3303c, applying the apramycin-DsRed2 positive selection and a KatG-dependent isoniazid counterselection strategy (17). The successful deletion of *leuB* was verified by PCR and confirmed by Southern blot analysis (Fig. 2a and b) as well as whole-genome sequencing (Fig. S1). Phenotypic characterization revealed that the *leuB* deletion mutant was auxotrophic for leucine, exhibiting growth only on agar plates supplemented with leucine (Fig. 2c). The *leuB* gene is part of a two-gene operon, where *serA* is the upstream gene and *leuB* the downstream gene. A complemented mutant strain was generated by transforming *M. abscessus* Δ*leuB* with the integrative plasmid pMV361-Apr-*leuB*, which contains the two-gene operon (*serA* and *leuB*) as well as the native promoter. Initial selection was done on plates containing apramycin while being supplemented with L-leucine. At the genetic level, complementation was confirmed by PCR and Southern blot analysis (Fig. 2a and b). Phenotypically, the complemented strain restored growth on leucine-free medium, consistent with the functional complementation of *leuB* (Fig. 2c). The complemented mutant strain is henceforth referred to as *M. abscessus* Δ*leuB::leuB*.

### Leucine auxotrophic selection marker reduces the overall background after transformation

The generation of the *leuB* deletion mutant and the successful genetic complementation enabled us to compare conventional antibiotic selection using apramycin with selection based on the restoration of leucine prototrophy. To this end, we used the complementation plasmid pMV361-Apr-*leuB*, which carries a functional copy of the disrupted *leuB* gene alongside an apramycin (Apr) resistance cassette. As controls, we included the pMV361-Apr vector backbone containing only the apramycin resistance cassette and the polyG-Apr vector backbone (which also confers apramycin resistance). These plasmids were transformed into both the parental *M. abscessus* ATCC 19977 strain (WT) and the Δ*leuB* mutant. Following electroporation, the cells were recovered in leucine-supplemented medium and plated on 7H10 agar, containing either apramycin (50 mg/L) or apramycin and L-leucine (200 mg/L), as well as on LB agar containing apramycin and L-leucine.

Comparing the transformation of *M. abscessus* WT and the leucine-auxotrophic Δ*leuB* mutant revealed profound differences in both transformation efficiency and background growth across antibiotic and leucine-based selection. The WT strains transformed with the apramycin-resistance plasmids pMV361-Apr, pMV361-Apr*-leuB*, and polyG-Apr displayed transformation rates consistently in the 4–9  ×  10³ CFU µg⁻¹ (Table 1). These transformation efficiencies were accompanied by substantial spontaneous background growth ranging from 11% to 20%, depending on the plasmid and plating condition (e.g., on 7H10–Apr background levels of 12.5% ± 3.3% for pMV361-Apr and 17.4% ± 7.4% for polyG-Apr were observed). In contrast, the Δ*leuB* strain showed minimal residual background growth (≤0.09%) when grown either under leucine-supplemented conditions or leucine-free conditions when functional complementation of *leuB* is required (Table 1), indicating a drastic reduction in non-specific colony formation compared with WT.

**TABLE 1:** Transformation efficacy (TE) and background (BG) of *M. abscessus* under selective conditions^*a*^

Strain	Plasmid	LB Apr Leu		7H10 Apr		7H10 Apr Leu		7H10	
		TE	BG	TE	BG	TE	BG	TE	BG
WT	pMV361-Apr^R^	9.4 × 10^3^ ± 3.4 × 10^3^	11.2 ± 5.2	6 × 10^3^ ± 1.8 × 10^3^	12.5 ± 3.3	7.5 × 10^3^ ± 2.1 × 10^3^	11.2 ± 4.1	n.d.	n.d.
Δ*leuB*		1.7 × 10^5^ ± 3.6 × 10^4^	0.06 ± 0.05	0 ± 0	0	1.2 × 10^5^ ± 4.0 × 10^4^	0.07 ± 0.07	0 ± 0	0
WT	pMV361-*leuB*	9.2 × 10^3^ ± 1.6 × 10^3^	11.0 ± 4.2	6.8 × 10^3^ ± 3.2 × 10^3^	11.9 ± 5.0	6.3 × 10^3^ ± 4.0 × 10^2^	13.0 ± 2.7	n.d.	n.d.
Δ*leuB*		1.6 × 10^5^ ± 6.8 × 10^4^	0.07 ± 0.07	1.1 × 10^5^ ± 3.7 × 10^4^	0	1.1 × 10^5^ ± 3.7 × 10^4^	0.09 ± 0.08	1.2 × 10^5^ ± 5.3 × 10^4^	0
WT	polyG-Apr^R^	5.8 × 10^3^ ± 1.5 × 10^3^	17.3 ± 7.2	4.5 × 10^3^ ± 2.1 × 10^3^	17.4 ± 7.4	3.8 × 10^3^ ± 1.2 × 10^3^	20.8 ± 2.1	n.d.	n.d.
Δ*leuB*		1.2 × 10^5^ ± 2.5 × 10^4^	0.07 ± 0.06	0 ± 0	0	1.3 × 10^5^ ± 2.2 × 10^4^	0.05 ± 0.05	0 ± 0	0

^*a*^ TE (in CFU/µg) and BG (%) were calculated based on CFU of WT/Δ*leuB* transformed without DNA. Apr, apramycin; Apr^R^, *aac(3)IV*, Apr resistance; Leu, L-leucine; n.d., not done.

To quantitatively compare transformation efficiencies (TE) while accounting for differences in background growth (BG), CFUs were normalized as log_10_(TE + 1) − log_10_(BG + 1), followed by two-way ANOVA, including genotype and growth condition as factors. Across all three plasmid types, a significant effect of strain genotype and plating condition, as well as a significant interaction between both factors, was observed (*P* < 0.001). Under non-selective conditions (7H10), WT exhibited confluent growth exceeding the quantifiable range, whereas the Δ*leuB* strain failed to grow, preventing meaningful statistical comparison. Similarly, under selective conditions lacking leucine supplementation (7H10–Apr), no transformants were detected for the Δ*leuB* strain, while WT retained measurable transformation activity, highlighting the strict dependence of the knockout mutant on leucine availability. Strikingly, under permissive conditions (7H10–Apr–Leu and LB–Apr-Leu), the Δ*leuB* strain consistently exhibited a significantly higher normalized transformation efficiency compared with WT across all plasmids tested. This increase amounted to approximately 2 log units (~100-fold) and was highly significant in all pairwise comparisons between WT and Δ*leuB* (Bonferroni post test, *P* < 0.001), seen in Fig. S2.

Notably, when Δ*leuB* was transformed with pMV361-Apr-*leuB* and plated on 7H10, robust transformant recovery (1.2  ±  0.53  ×  10⁵ CFU µg⁻¹, Table 1) was observed in the complete absence of antibiotic selection. While background growth remained strictly absent for all plasmids lacking *leuB* under the same condition (Table 1).

Together, these data demonstrate that leucine auxotrophy provides a highly stringent and virtually background-free selection system in *M. abscessus*, outperforming traditional antibiotic selection. Furthermore, complementation of Δ*leuB* enables efficient recovery of transformants without antibiotic pressure, highlighting the potential of this system for high-fidelity genetic manipulation.

### Construction of second-generation vectors for genetic manipulation of *M. abscessus*

**Fig 3:**
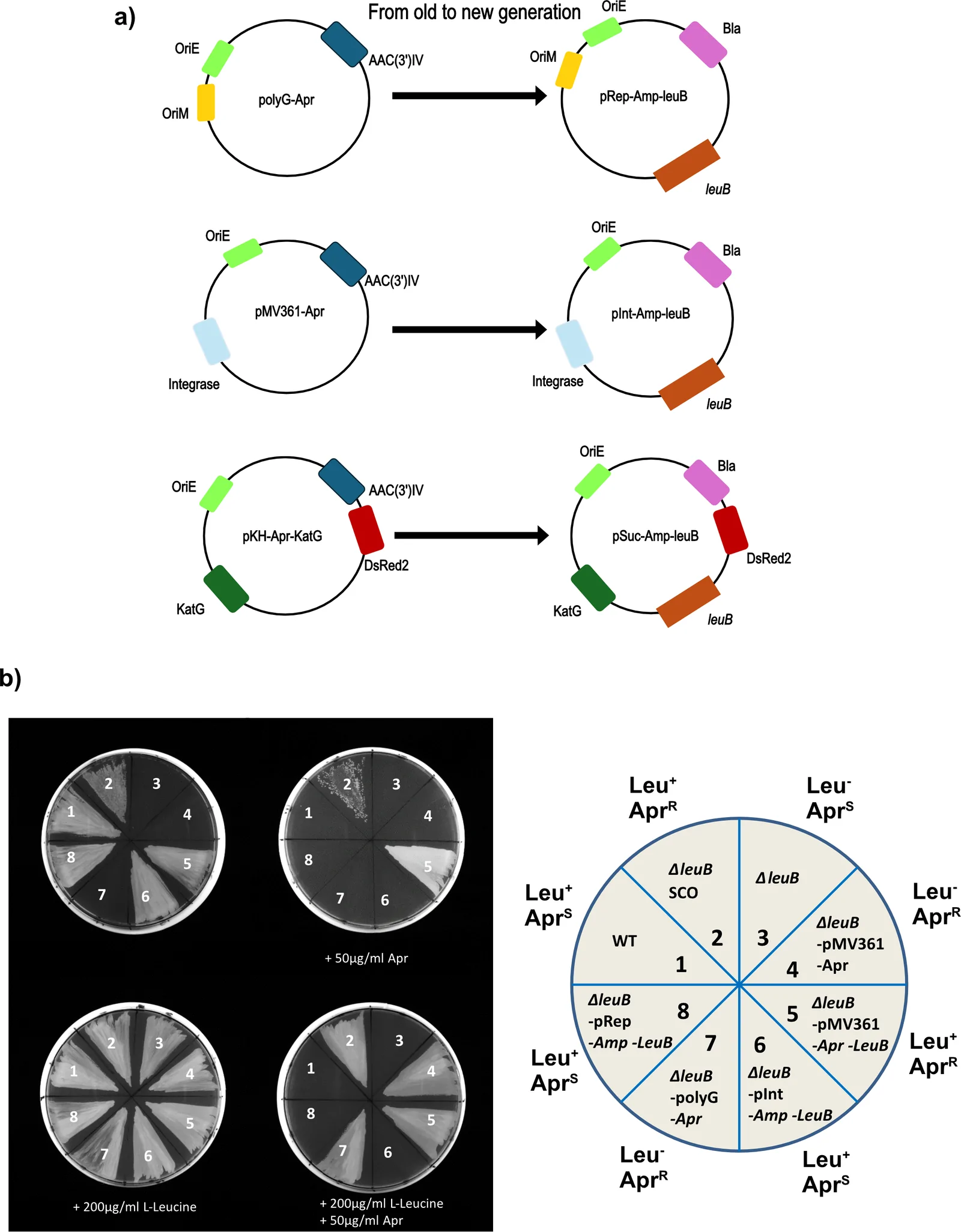
Phenotypic characterization of transformants with the second generation of vectors using leucine auxotrophy. (**a**) The old vector on the left versus the new vector on the right; we exchanged the apramycin resistance cassette for ampicillin resistance cassette and added leuB to complement and restore the leucine prototrophy. (**b**) Growth of leucine auxotroph *M. abscessus* transformed with new and old vectors on different agar plate. From left top to bottom right are following conditions depicted: 7H10, 7H10 with apramycin, 7H10 with L-leucine, and 7H10 with apramycin and L-leucine.

Following proof-of-concept study, we developed a set of second-generation vectors exploiting leucine auxotrophy as a positive selection marker. A key motivation was to reduce reliance on aminoglycoside selection, which is known to induce the *whiB7* regulon, a central regulator of antibiotic stress responses in *M. abscessus*. Although transient induction of *whiB7* does not necessarily interfere with downstream assays once selective pressure is removed, exposure to inducing antibiotics during strain construction or maintenance may continue to affect gene expression profiles and influence subsequent phenotypic analyses. By replacing aminoglycoside-based selection with leucine auxotrophy, this potential confounding source can be minimized. Based on this rationale, we constructed a new suite of plasmids: pRep-Amp-*leuB*, a replicative vector containing an ampicillin resistance cassette (for selection in *E. coli*) and the *leuB* gene, designed to replace the original polyG-Apr vector; pInt-Amp-*leuB*, an integrative vector containing an ampicillin resistance cassette, integrase, *attP* phage integration site, and *leuB*, serving as a substitute for pMV361-Apr; and pSuc-Amp-*leuB*, a suicide vector backbone for targeted gene deletion, carrying *leuB* for positive selection, along with *DsRed2* (red fluorescent protein) and *katG* as a negative selection marker as a substitute for pKH-Apr-KatG vector (Fig. 3a).

### Phenotypic characterization of leucine auxotrophic *M. abscessus* transformants

To functionally validate the constructs, the newly developed pRep and pInt vectors were transformed into an *M. abscessus* Δ*leuB* strain, alongside their apramycin-based predecessors (polyG-Apr, pMV361-Apr and pMV361-Apr-*leuB*, respectively). Transformants were plated on various 7H10 agar, with apramycin, with L-leucine, and with apramycin and L-leucine, respectively.

Figure 3b illustrates the growth of the different transformants on the respective selective agar plates: #1 represents the parental *M. abscessus* strain ATCC 19977, which grows on 7H10 and on 7H10 supplemented with leucine, reflecting its apramycin sensitivity (Apr^S^) and leucine prototrophy (Leu^+^). #2 corresponds to the *leuB* single cross-over strain generated by the first homologous recombination of the deletion plasmid pKH-Apr-DsRed2-KatG-MAB_3303c. This strain grows on all media, consistent with apramycin resistance (Apr^R^) and leucine prototrophy (Leu^+^). #3 shows the *MABΔleuB* strain, which requires leucine supplementation for growth. Accordingly, growth is observed only on 7H10 agar supplemented with leucine (Apr^S^, leucine auxotroph = Leu^-^). #4 and #5 depict the Δ*leuB* strain transformed with the previously used integrative plasmids pMV361-Apr and pMV361-Apr-*leuB*, respectively. In #4, growth is observed only on leucine-supplemented 7H10 agar, consistent with the leucine-auxotrophic background of the Δ*leuB* strain. In contrast, #5 shows growth on all agar plates, indicating restoration of leucine prototrophy through plasmid-borne *leuB*. #6 represents the Δ*leuB* strain transformed with the newly constructed integrative plasmid pInt-Amp-*leuB*. Growth is observed on 7H10 and leucine-supplemented 7H10 agar, but not on apramycin-containing plates, resembling the wild-type phenotype (Apr^S^, Leu^+^). #7 and #8 show the Δ*leuB* strain transformed with the previously used replicative vector polyG-Apr and the newly constructed replicative vector pRep-Amp-*leuB*, respectively. While the strain in #7 grows exclusively on leucine-supplemented agar (Apr^R^, Leu^−^), the strain in #8 grows on both 7H10 and leucine-supplemented 7H10 agar (Apr^S^, Leu^+^), similar to the parental strain.

Taken together, transformants carrying second-generation vectors did not grow on apramycin-containing plates (#6 and #8), confirming the absence of an aminoglycoside resistance cassette. In contrast, these strains showed robust growth on standard leucine-free 7H10 agar, indicating successful complementation of the *leuB* deletion. Conversely, transformants harboring vectors lacking a functional *leuB* grew only on L-leucine–supplemented plates, again validating the leucine-dependent selection system.

### Construction of *M. abscessus* deletion mutants involved in aminoglycoside resistance

We previously described genes involved in innate aminoglycoside resistance (25). Deletion of the *aac(2′*) encoding for an acetyltransferase in *M. abscessus* confers resistance to tobramycin, kanamycin B, dibekacin, and gentamicin C, whereas the deletion of *eis2* encoding the intracellular survival protein 2, also an acetyltransferase, confers resistance to capreomycin, hygromycin B, amikacin, and kanamycin B. To further challenge the leucine auxotrophy as a strong selection marker, we intended to generate deletion mutants of *aac(2*′) (MAB_4395) and *eis2* (MAB_4532c) in the *ΔleuB* strain. We constructed pSuc-derived suicide vectors (pSuc-Amp-*leuB*-Δ*aac(2*′), pSuc-Amp-*leuB*-Δeis2, respectively) that target the corresponding genes and then transformed these vectors into an *M. abscessus ΔleuB*. Rather than using apramycin to select transformants (17), we employed leucine prototrophy as the selective marker. Following PCR confirmation, the single cross-over transformants were subjected to KatG counterselection on agar plates supplemented with leucine and isoniazid. The deletion mutants were verified by PCR, Southern blot analysis (Fig. S3), and whole-genome sequencing (Fig. S4). The genetic characterization for vector-based markers was followed by phenotypic characterization on selective agar plates (Fig. S5). This process yielded the *M. abscessus* strains Δ*leuB*Δ*aac(2′)* [*Mab*Δ*leuB*Δ*aac(2*′)] and Δ*leuB*Δ*eis2* (*Mab*Δ*leuB*Δ*eis2*). The deleted genes of interest [*aac(2′*) and *eis2*] were then complemented using pInt-Amp-*leuB-aac(2′*) and pInt-Amp-*leuB-eis2*, respectively.

### Drug susceptibility testing of newly constructed *M. abscessus* deletion mutants

The susceptibility of *Mab*∆*leuB*∆*aac(2*′), *Mab*Δ*leuB*Δ*eis2* toward kanamycin B, amikacin, kanamycin A, hygromycin B, and, as a control unaffected by Eis2 or AAC(2′), apramycin, was tested in CAMH-Broth and compared with *Mab*Δ*leuB*, *Mab*∆*leuB*∆*aac(2*′), and *Mab*∆*leuB*∆*eis2* complemented with vector backbone pInt-Amp-leuB. Growth was visually determined on day 3, 5, 7, and after 12 days of incubation. Table 2 shows the median MIC value at day 5; MIC values for all time points are found in the supplement (Fig. S6).

**TABLE 2:** MICs for newly generated *M. abscessus* deletion mutants^*a*^

Compound	MIC (mg/L)				
	*∆leuB*::*leuB*	*∆leuB∆aac(2′)*::*leuB*	*∆leuB∆aac(2′)*::*leuB,aac(2*′)	*∆leuB∆eis2*::*leuB*	*∆leuB∆eis2*::*leuB,eis2*
KanB	8	0.125	8	2	8
Amk	2	2	2	0.25	2
KanA	1	1	1	0.25	1
HygB	>256	>256	>256	16	256
Apr	0.5	0.5	0.5	0.5	0.5

^*a*^ Depicted values are the medians on day 5. For the complete overview of MIC results from three independent experiments at days 3, 5, 7, and 12, see Fig. S6. KanB, kanamycin B; Amk, amikacin; KanA, kanamycin A; HygB, hygromycin B; Apr, apramycin.

The deletion and complementation of *leuB* had no influence on overall susceptibility; the values are similar to those we previously published (25) for the *M. abscessus* ATCC 19977. Comparing the Δ*leuB::leuB* strain with the Δ*leuBΔaac(2′)::leuB* strain, we observe a 64-fold reduction of the MIC for kanamycin B. For the Δ*leuBΔeis2::leuB* strain, we observe a reduction of MIC for kanamycin B (fourfold), amikacin (eightfold), kanamycin A (fourfold), and hygromycin B (>16-fold). Comparing the effect of Δ*aac(2*′) and Δ*eis2*, we notice that AAC(2′) specifically increases the MIC of kanamycin B, while Eis2 exhibits a broader substrate spectrum, capable of increasing MICs of several aminoglycosides. By complementing the Δ*aac(2*′) strain with pInt-Amp-leuB-aac(2′) as well as the Δ*eis2* strain with pInt-Amp-leuB-eis2, respectively, we restored the observed phenotypes back to the initial level of the Δ*leuB::leuB* (same as WT) level. The susceptibility of the control drug apramycin was not affected by either AAC(2′) or Eis2. The obtained MIC values for the *aac(2*′) and *eis2* deletion mutants are in accordance with our previously published data (25).

### Deletion of glycopeptidolipid (GPL) biosynthesis genes using leucine auxotrophy-based selection

**Fig 4:**
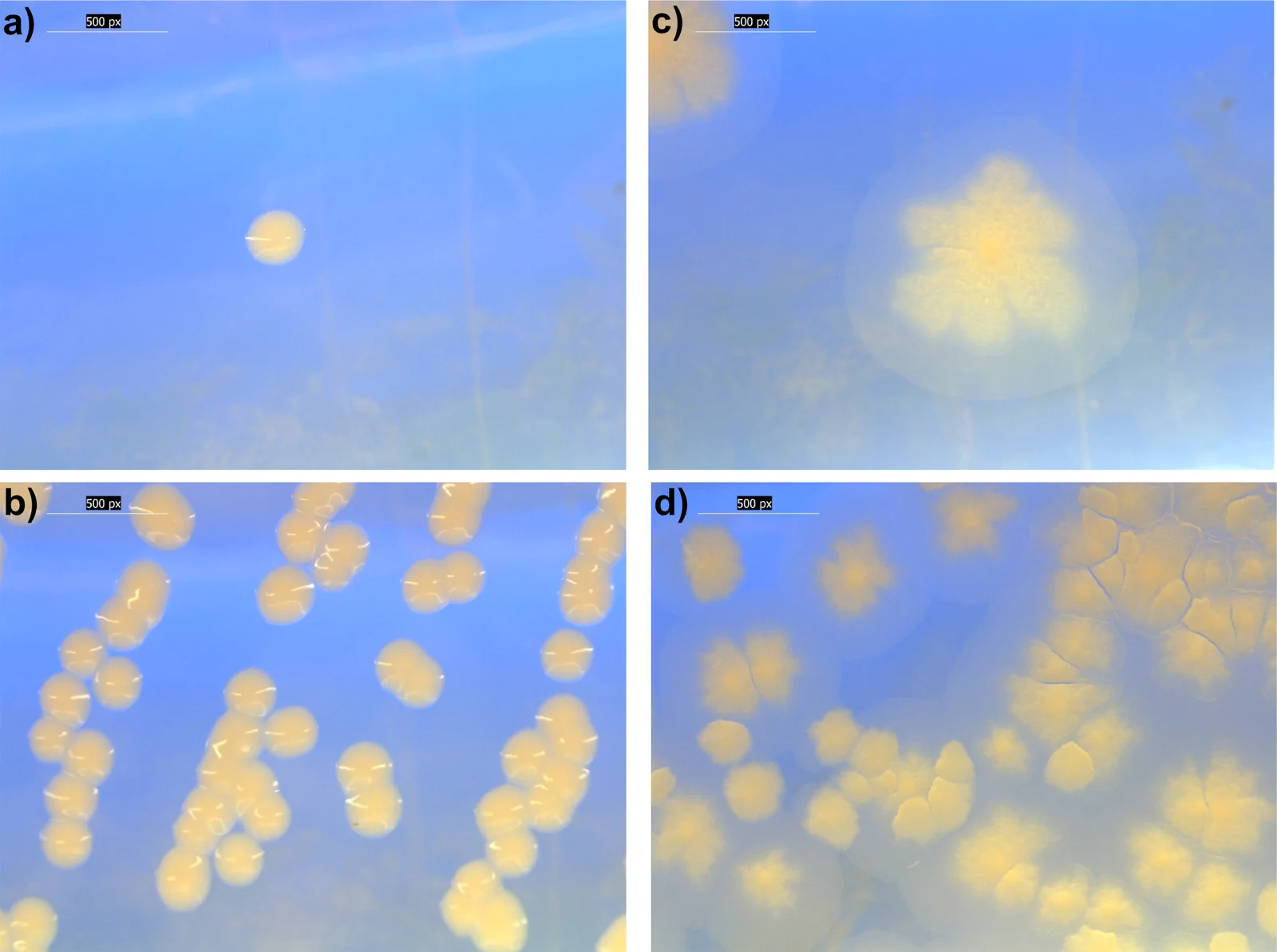
Morphology of GPL deletion mutant. (**a and b**) The parental *M. abscessus* ∆*leu* strain as a single colony and group image, respectively, which shows a “smooth” morphotype. (**c and d**) The ∆GPL mutant, lacking the glycopeptidolipid synthesis cluster, resulting in a “rough” morphotype. Images were taken after 6 days of incubation at 37°C with a Leica M80/MC170 HD microscope. 500 px corresponds to 4.27 mm.

In a clinical context, the transition from the smooth to the rough morphotype in *M. abscessus* is associated with increased virulence, enhanced intracellular survival, and resistance to phagocytosis (33). Smooth variants are usually found in early-stage infections and are characterized by GPL-mediated sliding motility and biofilm formation, whereas rough variants are prevalent in chronic infections and associated with severe pulmonary disease progression (33). To show the robustness of leucine auxotrophy as a selection system that goes beyond antibiotic resistance, we targeted the glycopeptidolipid biosynthesis locus in *M. abscessus*. This locus is essential for maintaining the smooth colony morphotype. Using the ∆*leuB* background strain (Fig. 4a and b), we constructed the suicide vector pSuc-Amp-LeuB-ΔGPL for deleting the entire GPL operon (MAB_4099c–MAB_4097c, deletion of 18 kbp), which encodes two non-ribosomal peptide synthases (*mps1* and *mps2*) and an integral membrane protein (gap) required for GPL translocation. We employed leucine prototrophy as the primary positive selective marker. Following PCR confirmation, the SCO transformants were subjected to KatG counterselection in presence of leucine. The resulting strain, *M. abscessus*∆*leuB* ∆*mps1-mps2*-gap (further *Mab*∆*leuB*∆*GPL*), exhibited a rough colony morphotype on leucine-supplemented 7H10 agar (Fig. 4c and d), consistent with the loss of surface GPLs. This deletion was confirmed by PCR and whole-genome sequencing (Fig. S7), as well as by inspecting the morphotype on agar plates. Our results demonstrate that leucine auxotrophy enables the efficient and antibiotic-free genetic manipulation of clinically relevant loci in *M. abscessus*.

## DISCUSSION

In this study, we present a novel genetic manipulation system and toolbox for *M. abscessus* that is based on leucine auxotrophy. This system offers a robust, antibiotic-free alternative for positive selection. This approach tackles several key issues in mycobacterial genetics, such as high background levels during transformation and the unintended activation of antibiotic stress responses, including the *whiB7* regulon (24). This study demonstrates that the apramycin-based transformation of *M. abscessus* ATCC 19977 results in transformation efficiencies that range from 10³ to 10⁴ CFU µg⁻¹, which is consistent with the previously reported efficiencies for this organism. Indeed, *M. abscessus* has historically been considered challenging to transform due to its restrictive cell envelope and active restriction–modification systems. Published studies describe transformation efficiencies as highly variable but generally low, often substantially below those achieved in *M. smegmatis* or *M. tuberculosis,* with plasmid mass, selection marker, and electroporation conditions being critical determinants of success (34, 35).

Quantitative analysis incorporating normalization of transformation efficiency (TE) to background growth (BG) [log_10_(TE + 1) − log_10_(BG + 1)] revealed that the Δ*leuB* mutant exhibits a marked increase in apparent transformation efficiency under permissive conditions. Specifically, two-way ANOVA demonstrated a highly significant effect of genotype and growth condition, as well as a significant interaction between both factors (*P* < 0.001). Post-hoc comparisons confirmed that under leucine-complemented conditions (7H10-Apr-Leu and LB-Apr-Leu), the Δ*leuB* mutant consistently displayed an approximately 2-log (~100-fold) higher normalized transformation efficiency compared with WT (Bonferroni posttest, *P* < 0.001). This effect was reproducible across all tested plasmids, indicating that it reflects a general physiological property of the mutant rather than a plasmid-specific phenomenon. Importantly, under non-permissive conditions lacking leucine supplementation (7H10-Apr), the Δ*leuB* strain failed to yield transformants, further emphasizing the strict dependence of this mutant on leucine availability. This conditional growth defect highlights a fundamental limitation of antibiotic-based selection systems, where background growth and antibiotic tolerance can obscure true transformation events.

A significant benefit of the leucine-based selection system is its remarkably low background level. The spontaneous background frequencies observed in the WT strain following apramycin selection ranged from 11% to 20%, whereas the Δ*leuB* strain demonstrated virtually background-free selection (≤ 0.09%) when selecting for leucine prototrophy. This high degree of selection stringency likely contributes to the observed increase in normalized transformation efficiency and underscores the advantage of auxotrophic selection systems in minimizing false-positive colony formation.

Aminoglycoside antibiotics frequently permit low-level spontaneous resistant colonies or non-specific growth of *M. abscessus*, as has been documented in multiple studies addressing transformation challenges in fast-growing NTMs (34, 35). In contrast, the auxotrophy-based system described here effectively eliminates background growth, thereby improving the reliability and interpretability of transformation assays. In this context, the transformation efficiencies observed for the Δ*leuB* mutant, which reach approximately 10⁵ CFU µg⁻¹ under permissive conditions, represent an enhancement of one to two orders of magnitude compared with WT under matched conditions. While recent advancements such as ultra-high field electroporation have yielded improvements in transformation efficiency, these approaches require specialized instrumentation and extensive optimization. The leucine auxotrophy-based system presented here achieves comparable improvements in effective transformation output under standard electroporation conditions, without the need for specialized equipment or protocol modification (34, 35).

We specifically targeted *leuB*, which encodes isopropyl malate dehydrogenase, because of its central role in leucine biosynthesis (28). Although leucine auxotrophy is commonly engineered by deletion of *leuCD* (36, 37), we chose to delete *leuB* to obtain a leucine-auxotrophic strain. *leuB* encodes an essential, non-redundant enzyme in the leucine biosynthesis pathway, and its deletion results in a stable and well-defined auxotrophic phenotype. Targeting a single gene minimizes genetic perturbation and reduces the risk of polar or pleiotropic effects associated with multigene deletions such as *leuCD*. In addition, retention of the upstream steps of the pathway avoids potential accumulation of aberrant intermediates and facilitates clean genetic complementation, making *leuB* deletion a suitable and controlled strategy for generating leucine auxotrophy. Of note, the complementation vector still carries an ampicillin selection marker, which however is only used in *E. coli*. Overall, the leucine auxotrophy system improves the efficiency of genetic manipulation of *M. abscessus* and reduces reliance on antibiotics during cloning. This supports antibiotic stewardship and minimizes the environmental impact of antibiotic use in research.

For complementation, the two-gene operon consisting of *serA* (upstream), *leuB* (downstream), and its native promoter (upstream) was introduced. The overlapping genomic organization of *serA* and *leuB* suggests that their expression is co-regulated. Consistent with this, attempts to complement Δ*leuB* using *leuB* alone were unsuccessful, indicating that proper expression depends on the promoter of the upstream gene. While *serA* is known to be associated with metabolic phenotypes, we did not observe any additional phenotypic effects beyond restoration of leucine prototrophy under the conditions tested. Although we cannot completely exclude subtle contributions from *serA* or local regulatory effects, the observed phenotype is most parsimoniously explained by restoration of *leuB* function.

The *whiB7* regulon represents a central regulatory node controlling intrinsic resistance to multiple ribosome-targeting antibiotics in *M. abscessus (23*). Upon exposure to macrolides, aminoglycosides, and additional stressors, *whiB7* is induced and activates a broad set of downstream genes, including *erm(41), eis2,* and ABC-F ribosome-associated rescue factors (22, 24). These collectively contribute to inducible resistance and cross-protection against structurally diverse antibiotics. While many genetic workflows rely on transient antibiotic selection followed by propagation under non-selective conditions, it is important to consider that prior exposure to inducing antibiotics may influence bacterial physiology and transcriptional states. Induction of stress-response regulons, such as whiB7, may persist or alter baseline expression of resistance-associated pathways, potentially affecting the interpretation of phenotypic assays under certain experimental conditions. In this context, the use of leucine auxotrophy as a selection system provides a distinct advantage by eliminating the need for antibiotic pressure during strain construction and maintenance. This reduces the likelihood of unintended regulatory activation and enables more controlled assessment of bacterial phenotypes, particularly in studies focusing on antimicrobial susceptibility, stress responses, or gene function. Recent work by Hurst-Hess et al*.* used ChIP-Seq and RNA-Seq and mapped over 50 WhiB7-binding sites in clinical and laboratory strains. The study showed that WhiB7 acts exclusively as a transcriptional activator at promoters dependent on either σ^A^ or σ^B^, and that multi-resistance emerges as the cumulative effect of both major and minor effectors in the regulon. Studies of clinical isolates also reveal that the dynamics of *erm(41*) and *whiB7* expression differ between isolates, with azithromycin inducing *erm(41*) more rapidly than clarithromycin *in vivo*. We do not imply that antibiotic-based selection universally biases susceptibility testing; rather, our approach minimizes a potential source of variability and provides an alternative strategy for experiments requiring tightly controlled physiological conditions. By eliminating this confounding factor induced by antibiotic selection, we can potentially obtain more accurate phenotypic analysis of recombinant strains (deletion mutant, complemented mutant), particularly with regard to MIC testing and drug susceptibility profiling. Given the clinical importance of *M. abscessus* as a multidrug-resistant pathogen, this refinement is potentially significant for translational research and therapeutic development (38). In addition to improving selection fidelity, our system also reduces the volume of heat-stable antibiotics required for routine cloning and selection. This lowers the environmental burden associated with antibiotic use in research. By replacing antibiotics with metabolic complementation, our system adheres to the principles of green microbiology (39), thereby reducing the ecological footprint of microbial genetics research. This genetic toolbox, which includes the replicative (pRep-Amp-*leuB*), integrative (pInt-Amp-*leuB*), and suicide (pSuc-Amp-*leuB*) vectors, provides a flexible and versatile platform for generating deletion mutants, complementing those knockout mutants, and conducting functional genomic studies in *M. abscessus*.

Not only did we target aminoglycoside resistance genes for deletion, but we also targeted the glycopeptidolipid biosynthesis locus. This allowed us to induce a morphotype switch from smooth to rough by deleting a 19 kbp large genomic segment, a transition that has profound implications for pathogenicity. The smooth morphotype is characterized by the presence of surface GPLs, and is associated with sliding motility, biofilm formation, and reduced immune activation (40, 41). Conversely, the rough morphotype lacks GPLs, exhibits enhanced aggregation, and is associated with increased virulence, including improved intracellular survival and resistance to phagocytosis by macrophages (42). This morphotypic switch is not merely a phenotypic curiosity but rather reflects a critical adaptation during chronic infection. Rough variants are frequently isolated from patients with advanced pulmonary disease and are associated with poor clinical outcomes (43, 44). Therefore, the ability to genetically engineer this transition independently of aminoglycoside resistance markers opens new avenues for studying *M. abscessus* pathogenesis under clinically relevant conditions. Of note, recent research indicates differences in the essential gene set of smooth and rough strains (45).

An additional advantage of the selection system described here is its potential applicability beyond *M. abscessus*. The vectors used in this study contain a mycobacterial origin of replication (OriM) and chromosomal attachment site (*att*P), respectively, which is known to support replication and integration across a range of mycobacterial species. This suggests that, in principle, the auxotrophy-based selection system could be extended to other mycobacteria, provided that suitable leucine auxotrophic strains can be generated. Leucine auxotrophs have previously been described in related species such as *M. tuberculosis* and *Mycobacterium bovis* BCG (46), indicating that this metabolic dependency can be engineered across different members of the genus. Consequently, the combination of OriM-based/*att*P vectors with auxotrophic selection represents a broadly transferable genetic toolkit. This approach may be particularly advantageous in the context of drug susceptibility testing. Unlike antibiotic resistance markers, which can influence bacterial physiology and activate stress response pathways, such as the whiB7 regulon, auxotrophic complementation provides a selection system that is independent of antimicrobial pressure. As a result, this strategy minimizes confounding effects on intrinsic drug susceptibility and enables more accurate assessment of antibiotic responses.

Looking to the future, leucine auxotrophy-based selection provides a basis for more advanced genetic applications, such as Tn mutagenesis, CRISPR-based editing, high-throughput mutagenesis. A potential limitation of the leucine auxotrophy-based system is its restricted applicability in *in vivo* infection models. Previous studies in *M. tuberculosis* have demonstrated that leucine auxotrophic mutants are unable to replicate in macrophages and are severely attenuated in animal models, likely due to limited availability of leucine within host tissues (32, 46, 47). Consequently, such mutants are often rapidly cleared or fail to establish productive infections *in vivo*. These findings suggest that the *ΔleuB* strain described here will primarily be suitable for *in vitro* and *ex vivo* applications, including macrophage infection studies and genetic manipulation, but may have limited utility for studying virulence or persistence in animal models. Alternatively, complementation of the mycobacterial *leuB* strains with pInt-Amp-*leuB* may also rescue the *in vivo* phenotype.

Our approach supports the dual goals of scientific rigor and environmental responsibility, addressing longstanding challenges in mycobacterial genetics while enabling more sustainable and precise research.

In summary, the genetic toolbox developed in this study is a significant step forward in mycobacterial genetics. It provides a robust and sustainable alternative to traditional antibiotic-based selection systems. By combining scientific rigor with environmental responsibility, this approach addresses key challenges in microbial genetic manipulation and contributes to more sustainable research practices. While it is currently only applicable to engineered ∆*leuB* strains and cannot be used directly with wild-type *M. abscessus*, the ATCC reference strain is widely used in research, and deletion or complementation of *leuB* does not further affect its phenotype. This supports the usefulness and reliability of the system.

## MATERIALS AND METHODS

### Bacterial strains and growing conditions

*Escherichia coli* strains were cultivated in LB broth at 37°C while shaking overnight. Antibiotics were added at the indicated concentrations when appropriate. *E. coli* XL1-Blue MRF was used for all cloning procedures. *M. abscessus* strains were routinely cultured in Middlebrook 7H9 liquid medium at 37°C with shaking or on Middlebrook 7H10 agar plates for solid growth and phenotypic assays. For auxotrophic selection and complementation experiments, 7H10 agar was used as the standard medium. Where indicated, media were supplemented with L-leucine (200 mg/L) to support growth of leucine auxotrophic strains. Leucine-free medium refers to 7H10 agar without L-leucine supplementation and was used to select for strains complemented with *leuB*. When required, apramycin was added to a final concentration of 50 mg/L. LB agar was used only for specific experimental steps, as a rich medium and supplemented with extra leucine where indicated, which is explicitly stated in the corresponding sections.

A list of all bacterial strains used in this study is provided in Fig. S8 and Table S1.

### Preparation of electrocompetent *M. abscessus*

A total of 400 mL of a dense *M. abscessus* culture (OD₆₀₀ = 0.4–0.8) was harvested by centrifugation at 4°C for 20 min. The supernatant was then discarded, and the cell pellet resuspended in 10% (vol/vol) ice-cold glycerol. This washing step was repeated several times, reducing the resuspension volume by half each time to progressively concentrate the cells. Washing continued until a final volume of 2 mL was reached. The resulting cell suspension was divided into 100-µL portions, snap-frozen in liquid nitrogen, and stored at −80°C until used.

### Deletion and complementation of MAB_3303c (*leuB*) in *M. abscessus*

The flanking regions of Mab-leuB (MAB_3303c) were amplified by PCR using genomic DNA from *M. abscessus* ATCC 19977. The 5′ upstream and 3′ downstream fragments were generated using primers p1/p2 and p3/p4, respectively, with *MunI/PvuI* and *PvuI/MunI* restriction sites incorporated as overhangs. The two PCR fragments (approx. 1.5 kbp each) were then assembled into the linearized deletion vector pKH-Apr-DsRed2-KatG (digested with *MunI* and *PvuI*) using Gibson Assembly. The resulting suicide plasmid, pKH-Apr-DsRed2-KatG-MAB3303c, carries an apramycin resistance cassette [*aac(3′)IV*] for positive selection, as well as *katG*, which confers sensitivity to isoniazid (a prodrug requiring KatG activation), and acts as a counterselectable marker. The recombinant plasmid was then transformed into *M. abscessus* ATCC 19977 electrocompetent cells. For selection steps involving the Δ*leuB* strain, media were supplemented with L-leucine (200 mg/L), unless otherwise specified. After 5 h of recovery at 37°C with shaking, the cells were diluted and plated onto LB agar containing apramycin (50mg/L) and supplemented with L-leucine (200mg/L). Transformants undergoing a single homologous recombination event (single crossover, SCO) were identified by PCR screening. These SCO transformants were then subjected to counterselection on LB agar plates containing isoniazid (32 mg/L) and L-leucine (200 mg/L) to select for double crossover (DCO) events through intra-molecular recombination. Individual colonies were screened by PCR for the deletion of *leuB*, and the genotype was confirmed by Southern blot analysis using a 0.5-kb *SexAI* 5′ *leuB* DNA probe as well as WGS (Fig. S1).

For complementation, the two gene operon consisting of *leuB* (MAB_3303c), *serA* (MAB_3304c), and its putative promoter was amplified by PCR from genomic DNA of *M. abscessus* ATCC 19977 using primers modified with a *KpnI* linker (p13/p14). The fragment was ligated to the linearized integrative vector pMV361-Apr via Gibson Assembly. pMV361-Apr carries the *int* and *attP* sites required for phage L5-mediated integration at the chromosomal *attB* locus (48, 49), as well as the *aac(3′)IV* that confers apramycin resistance. The pMV361-Apr-leuB construct was verified by Sanger sequencing and introduced into *M. abscessus* Δ*leuB* electrocompetent cells. Transformants were selected on LB agar containing apramycin (50 mg/L), and the integration of *leuB* at the *attB* locus was confirmed by PCR and Southern blot analysis. The inclusion of the whole operon was intended to preserve the native promoter as *leuB* and *serA* partially overlap and likely form an operon. Complementation attempts using *leuB* alone were not successful.

A comprehensive list of plasmids and primers used in this study is provided in Tables S2 and S3.

### Determination of transformation efficiency

To determine the transformation efficiency and selectivity of various plasmids, they were introduced into electrocompetent *M. abscessus* ATCC 19977 wild type (WT) and leucine auxotrophic (Δ*leuB*) strains. Following electroporation, the cells were plated onto Middlebrook 7H10 agar that had been supplemented with either apramycin (50 mg/L) or L-leucine (200 mg/L), or both. In addition, cells were plated on LB agar containing apramycin (50 mg/L) and supplemented with L-leucine (200 mg/L). To compare apramycin- and LeuB-based selection, the following plasmids were used: pMV361-Apr, pMV361-Apr-*leuB,* and polyG-Apr. A total of 500 ng of plasmid DNA was transformed into 100 μL of electrocompetent cells using 4-mm cuvettes. Electroporation was performed using a Bio-Rad Gene Pulser with the following settings: 2,500 V, 25 μF capacitance, and 1,000 Ω resistance. Immediately after electroporation, 900 µL of pre-warmed Middlebrook 7H9 medium supplemented with L-leucine (200 mg/L) was added, and the cells were incubated for 5 h at 37°C with shaking at 1,000 rpm in a ThermoMixer C (Eppendorf). The recovered cells were then washed twice with phosphate-buffered saline (PBS, pH 7.4) and resuspended in 7H9 medium or LB medium, respectively (1-mL final volume). Serial 10-fold dilutions (1:10, 1:100, and 1:1,000) were prepared, and 100 μL of each dilution was spotted onto the selective 7H10 or LB agar plates described above. The plates were then incubated for 5 days at 37°C, and the colony-forming units (CFUs) were counted to determine the transformation efficiency according to equation 1:


(1) $$Transformation\;efficacy\;\left(\frac{CFU}{\mu g}\right)=\left(C\times Vt\right)/\left(Vp\times Df\times DNA\right)$$

where *C* is counted colonies, *Vt* is volume total, *Vp* is volume plated, *Df* is dilution factor, and DNA is transformed DNA in μg. For statistical analysis, transformation efficiency (TE) and background (BG) values were log-transformed after addition of a pseudo count of 1 to avoid undefined values:


(2) $$Score=\log_{10}\left(TE+\varepsilon \right)-\;\log_{10}\left(BG+\;\varepsilon \right)$$

where TE is transformation efficiency, BG is background, and *ε* = 1.

For each transformed vector (pMV361-Apr, pMV361-Apr-*leuB*, and polyG-Apr), a two-way ANOVA was performed to assess the effects of genotype (WT vs *leuB*) and growth condition (7H10, 7H10-Apr, 7H10-Apr-Leu, LB-Apr-Leu). Multiple comparisons between genotypes within each condition were conducted using Bonferroni’s post hoc test, as seen in Fig. S2. Confluent growth (lawn formation) was observed for WT under non-selective conditions (7H10). As these values exceeded the quantifiable range, they were conservatively approximated for statistical analysis. All statistical analyses were performed using GraphPad Prism (version 5).

### Generation of second-generation vectors exploiting leucine auxotrophy

The plasmid pRep-Amp-*leuB* was constructed as follows: the ampicillin resistance cassette was amplified by PCR from the pBluescript-Int vector (50) using the primer pair p22/p23 to introduce *HpaI* and *MunI* restriction sites, respectively. The *leuB* gene was amplified from the wild-type *M. abscessus* genomic DNA using primer pair p24/p25, which introduced *PcaI* and *HpaI* linkers, respectively. The *E. coli* plasmid origin of replication (OriE) was amplified from the polyG-Apr vector using the primer pair p26, which contains a *PcaI* linker, and p27. The mycobacterial origin of replication (Alori) was amplified from the polyG-Apr vector using the primer pair p28/p29; the p29 primer introduced a *MunI* linker. All PCR fragments were assembled using Gibson Assembly according to the manufacturer’s protocol. One microliter of the assembly mix was transformed into electrocompetent *E. coli XL1*-Blue cells. Electroporation was performed at 1,800 V, 2 μF, and 200 Ω using 1-mm cuvettes. After electroporation, 1 mL of pre-warmed LB medium was added, and the cells were incubated at 37°C for 40 min with shaking at 1,000 rpm. The transformed cells were plated onto LB agar containing 100 mg/L ampicillin. Plasmid DNA was then isolated from the resulting ampicillin-resistant colonies and verified by Sanger sequencing.

The plasmid pInt-Amp-*leuB* was constructed as follows: the ampicillin resistance cassette was amplified by PCR from the pBluescript-Int vector (50) using the primer pair p30/p31 *HpaI* linker for p31 fragment. The *leuB* gene was amplified from the wild-type *M. abscessus* genomic DNA using primer pair p32/p33 with *HpaI* linker for p33 fragment. The Integrase (int)*—E. coli* Origin of replication (OriE)*—attP* fragment was amplified from vector pMV361-Apr vector with primer pair p34/p35. All PCR fragments were assembled using Gibson Assembly according to the manufacturer’s protocol. One microliter of the assembly mix was transformed into electrocompetent *E. coli XL1*-Blue cells. Electroporation, selection, and plasmid verification were conducted as described above.

The plasmid pSuc-Amp-*leuB* was constructed as follows: the ampicillin resistance cassette was amplified by PCR from the pBluescript-Int vector (50) using the primer pair p36/p37 with *HpaI*/*MunI* linker, respectively. *leuB* was amplified from the wild-type *M. abscessus* genomic DNA using primer pair p38/p39 with *PcaI*/*HpaI* linker, respectively. *E. coli* Origin of replication was amplified from pMV361-Apr vector with primer pair p40/p41 with *Eco105I*/*Eco72I* linker, respectively. The *katG* gene was amplified from pSE vector with primer pair p42/p43 with *Eco72I*/*PcaI* linker, respectively. DsRed2 was amplified from plasmid pMSP12 with primer pair p44/p45 with*MunI*/*Eco105I* linker, respectively. All PCR fragments were assembled using Gibson Assembly according to the manufacturer’s protocol. One microliter of the assembly mix was transformed into electrocompetent *E. coli XL1*-Blue cells. Electroporation, selection, and plasmid verification were conducted as described above.

### Phenotypic characterization on selective agar plates

The growth of various *M. abscessus* strains harboring newly constructed or previously established vectors was visually assessed under different culture conditions. The following strains were included: wild-type (WT); single-crossover (SCO; pKH-Apr-*leuB*); Δ*leuB;* Δ*leuB::pMV361-Apr;* Δ*leuB::pMV361-Apr-leuB;* Δ*leuB::pInt-Amp-leuB;* Δ*leuB::polyG-Apr*; and Δ*leuB::pRep-Amp-leuB*. All strains were pre-cultured in Middlebrook 7H9 medium, supplemented with either L-leucine (200 mg/L) or apramycin (50 mg/L) as required. The cultures were then washed twice with phosphate-buffered saline (PBS, pH 7.4), and 10 μL of each cell suspension was streaked onto Middlebrook 7H10 agar plates, which were supplemented with either apramycin (50 mg/L), L-leucine (200 mg/L), or both. The plates were then incubated for 5 days at 37°C, and the colony growth was documented using a Bio-Rad ChemiDoc XRS imaging system. The same procedure was applied for phenotypic characterization of the following strains: Δ*leuB*, Δ*aac(2′),* SCO (pKH and pSuc vectors); Δ*leuB*Δ*aac(2′);* Δ*leuB*Δ*aac(2′)::pInt-Amp-leuB-aac(2′*), and vector backbone; Δ*leuB*, Δ*eis2*, SCO (pKH-Δeis2 and pSuc-Δeis2 vectors); Δ*leuB*Δ*eis2;* Δ*leuB*Δ*eis2::pInt-Amp-leuB-eis2,* and vector backbone. Representative results are shown in Fig. S5a and b, respectively.

### Deletion and complementation of MAB_4395 [*aac(2*′)] in *M. abscessus ∆leuB*

The plasmid pSuc-Amp-*leuB-*∆*aac(2*′) was constructed as follows: The flanking region of Mab-*aac(2*′) (MAB_4395) was amplified by PCR from pKH-Apr-DsRed2-Δ*aac(2*′) with primer pair p46/p47. The pSuc-Amp-*leuB* was linearized by *HpaI* overnight digestion at 37°C and purified with Qiagen gel purification kit. All fragments were assembled using Gibson Assembly according to the manufacturer’s protocol. One μL of the assembly mix was transformed into electrocompetent *E. coli XL1*-Blue cells. Electroporation was performed at 1,800 V, 2 µF, and 200 Ω using 0.1-mm cuvettes. After electroporation, 1 mL of pre-warmed LB medium was added, and the cells were incubated at 37°C for 40 min with shaking at 800 rpm. The transformed cells were plated onto LB agar containing 100 mg/L ampicillin. Plasmid DNA was then isolated from the resulting ampicillin-resistant colonies and verified by Sanger sequencing. This vector was transformed into *M. abscessus* ∆*leuB* electrocompetent cells, and after 5 h of recovery at 37°C shaking, cells were diluted and plated out on selective LB agar (200 mg/L L-leucine). Selected transformants, which underwent intermolecular recombination, were identified by PCR. Single crossover transformants were subjected to counterselection on LB agar plates containing isoniazid (32 mg/L) and L-leucine (200 mg/L). Single colonies were screened for deletion of *aac(2*′) by PCR, and the genotype was confirmed by Southern blot analysis with a 0.3-kbp *Van91I* 3′-*aac(2*′) DNA probe (Fig. S3a) as well as WGS (Fig. S4a).

The plasmid pInt-Amp-*leuB*-aac(2′) was constructed as follows: *aac(2*′), including the native promoter, was amplified by PCR from genomic DNA of *M. abscessus* ATCC 19977 using *HpaI* linker-modified primers (p52/p53). The pInt-Amp-*leuB* plasmid (carrying integrase and *attP* for integration at the mycobacterial phage L5 *attB* locus in the genome of *M. abscessus*) was linearized with *HpaI*. Vector assembly, electroporation, selection, and plasmid and genotype verification were conducted as described above.

### Deletion and complementation of MAB_4532c (*eis2*) in *M. abscessus ∆leuB*

The plasmid pSuc-Amp-*leuB-∆eis2* was constructed as follows: the flanking region of *eis2* (MAB_4532c) was amplified by PCR from pKH-Apr-DsRed2-*Δeis2* vector with primer pair p54/p55. The pSuc-Amp-*leuB* was linearized by *HpaI* overnight digestion at 37°C and purified with Qiagen gel purification kit. Vector assembly, electroporation, selection, and plasmid verification were conducted as described above. This vector was transformed into *M. abscessus* ∆*leuB* electrocompetent cells, and after 5 hours of recovery at 37°C shaking, cells were diluted and plated out on selective LB agar (200mg/L L-leucine). Selected transformants, which underwent intermolecular recombination, were identified by PCR. Single crossover transformants were subjected to counterselection on LB agar plates containing isoniazid (32 mg/L) and L-leucine (200 mg/L). Single colonies were screened for deletion of *eis2* by PCR, and the genotype was confirmed by Southern blot analysis with a 0.3-kbp *Van91I* 3′-*eis2* DNA probe (Fig. S3b) as well as WGS (Fig. S4b).

The plasmid pInt-Amp-*leuB-eis2* was constructed as follows: *eis2,* including the native promoter, was amplified by PCR from genomic DNA of *M. abscessus* ATCC 19977 using *HpaI* linker-modified primers (p60/p61). The pInt-Amp-*leuB* plasmid was linearized with *HpaI*. The two fragments were assembled using Gibson Assembly according to the manufacturer’s protocol. One microliter of the assembly mix was transformed into electrocompetent *E. coli XL1*-Blue cells. Vector assembly, electroporation, selection, and plasmid and genotype verification were conducted as described above.

### Drug susceptibility testing of *aac(2*′*)* and eis2 deletion mutant

Stock solutions of kanamycin B, amikacin, kanamycin A, apramycin, and hygromycin B (all from Sigma-Aldrich) were prepared by dissolving each antibiotic in sterile distilled water according to the manufacturer’s instructions. The solutions were filter-sterilized, divided into smaller quantities (aliquots), and stored at −20°C until use. MIC assays were performed in accordance with the guidelines of the Clinical and Laboratory Standards Institute (CLSI) and as previously described (17). Briefly, working solutions of the antibiotics were prepared by diluting the stock solutions in cation-adjusted Mueller–Hinton broth (CAMH-Broth; pH 7.4; Becton Dickinson, Switzerland) to twice the desired final concentration. Twofold serial dilutions were then prepared in sterile 96-well microtiter plates. Each plate included a positive growth control (no antibiotic) and a negative control (CAMH-Broth only). Bacterial inocula were prepared by transferring 3–4 colonies from freshly grown LB agar plates into 2 mL of 0.9% (wt/vol) NaCl using sterile cotton swabs. The suspensions were adjusted to a turbidity equivalent to a 0.5 McFarland standard, yielding a final inoculum density of 1–5 × 10⁵ CFU/mL after dilution in CAMHB. Each well contained a total volume of 100 μL. Inoculum titers were verified by plating serial dilutions on LB agar.

The plates were sealed with adhesive covers and incubated at 37°C. MIC readings were recorded on days 3, 5, 7, and 12 for *M. abscessus* strains. All assays were performed in triplicate to ensure reproducibility. Representative data are shown in Fig. S6.

### Construction of the pSuc-Amp-leuB-ΔGPL plasmid and generation of the ΔGPL mutant

The plasmid pSuc-Amp-*leuB-*Δ*GPL* was constructed as follows: The flanking regions of the GPL operon (MAB_4099c–MAB_4097c) were amplified by PCR using genomic DNA from *M. abscessus* ATCC 19977 as a template. The 5′ (upstream) and 3′ (downstream) fragments were generated using primer pair p62/63 respectively p64/65 containing *HpaI* restriction site overhangs. The plasmid pSuc-Amp-*leuB* was linearized by *HpaI* digestion overnight at 37°C, then purified using a Qiagen Gel Extraction Kit. Vector assembly, electroporation, selection, and plasmid verification were conducted as described above. The confirmed pSuc-Amp-*leuB-*Δ*GPL* plasmid was then transformed into *M. abscessus* Δ*leuB* cells, followed by selection, respectively counterselection, as described above. Individual colonies were screened for the deletion of the GPL operon by PCR, and successful gene deletion was confirmed by morphology on agar plates. In addition, we performed WGS analysis of the Δ*GPL* mutant (Fig. S7).

### Phenotypic characterization of the ΔGPL mutant

For phenotypic analysis, the colony morphology of *M. abscessus* Δ*leuB* Δ*GPL* was compared with that of the parental *M. abscessus* Δ*leuB* strain. Bacterial suspensions were streaked on leucine-supplemented 7H10 agar. After incubation for 6 days at 37°C, colony morphology was documented using a Leica M80/MC170 HD microscope.

## Data Availability

All the data supporting the findings of this study are available from the corresponding author upon request. Raw reads from WGS analysis for the used *M. abscessus* strains have been deposited at the NCBI under BioProject accession number PRJNA1436954.

## SUPPLEMENTAL MATERIAL

Supplemental material aem.00712-26-s0001.docx Fig. S1 to S8; Tables S1 to S3.

ASM does not own the copyrights to Supplemental Material that may be linked to, or accessed through, an article. The authors have granted ASM a non-exclusive, world-wide license to publish the Supplemental Material files. Please contact the corresponding author directly for reuse.
