# Supplementary material for: Leucine auxotrophic marker for genetic manipulation of Mycobacterium abscessus
Source: Appl Environ Microbiol. 2026 Jul 16;92(8):e00712-26. doi: 10.1128/aem.00712-26 (PMC13488338; doi:10.1128/aem.00712-26)
Supplement: Supplementary file 1 [file aem.00712-26-s0001.docx]

Supplementary Information For

**Leucine auxotrophic marker for genetic manipulation of
*Mycobacterium abscessus***

Tizian Griesser*, Petra Selchow, Peter Sander*

*To whom correspondence should be addressed. Email: [tizian.griesser@uzh.ch](mailto:tizian.griesser@uzh.ch) and [psander@imm.uzh.ch](mailto:psander@imm.uzh.ch)

This PDF file includes:

Supplementary Figures 1 to 8

Supplementary Tables 1 to 3

Figure 1: Whole genome sequence analysis of leuB. The genomic region around leuB of the ∆leuB mutant is depicted. Read coverage for leuB (MAB_3303c) is zero (gap 957 bp), corresponding to the deletion of this gene.

Figure 2: Effect of leuB deletion and growth conditions on transformation efficiency. Normalized transformation efficiency was calculated as log10(TE + 1) − log10(BG + 1), where TE represents transformation efficiency and BG background colony formation. Data are shown for three vectors in a) for pMV361-Apr in b) for pMV361-Apr-leuB and in c) for polyG-Apr across four growth conditions (7H10, 7H10-Apr, 7H10-Apr-Leu, and LB-Apr-Leu). Each point represents an independent biological replicate (n = 3), and horizontal lines indicate the mean. Statistical analysis was performed using two-way ANOVA followed by Bonferroni’s multiple comparisons test. Significant differences between WT and leuB mutant within the same condition are indicated (*** p < 0.001). Confluent growth (lawn formation) was observed for WT under non-selective conditions (7H10), preventing accurate quantification of colony-forming units. These values were conservatively approximated for visualization (>10^8^) but excluded from statistical comparisons. Across all constructs, the leuB mutant exhibited no growth under non-complemented selective conditions (7H10-Apr), while showing a consistent ~2-log increase in transformation efficiency compared to WT under permissive conditions (7H10-Apr-Leu and LB-Apr-Leu).

Figure 3: Southern blot analysis of aac(2’) and eis2 deletion in the leucine auxotrophic M. abscessus strain in comparison with the conventional method using apramycin for positive selection. **a)** Southern blot analysis for deletion of aac(2’). A 150 bp probe was designed complementary to 150 bp in the flanking region downstream of the aac(2’) gene and genomic DNA was digested with Van91I. Line 1 and line 6 depicts the ∆leuB strain, the expected band size is 2.5 kb. Line 2 depicts the SCO obtained with suicide vector pKH-aac(3’)-DsRed2-KatG-∆MAB_4395, expected band size 2.1 kb and 2.5 kb. Line 3 depicts the resulting double mutant after counterselection with isoniazid (M. abscessus ∆ leuB ∆ aac(2’)), expected band size is 2.1 kb. Line 4 depicts the complementation of the leuB, aac(2’) double deletion with aac(2’) (new vector pInt), expected band size is 2.1kb and 11.2 kb. Line 5 and 9 depict DIC Ladder II/III. Line 7 depicts the SCO obtained with new pSuc vector, expected band size is 2.1 kb and 2.5 kb, line 8 depicts the double deletion mutant, expected band size is 2.1 kb. **b)** Southern blot analysis for deletion of eis2 gene. A 366 bp probe was designed complementary to 366 bp in the flanking region downstream of the eis2 gene and genomic DNA was digested with Van91I. Line 1 depicts DIC Ladder II and line 6 DIC Ladder III. Line 2 and 7 depicts the ∆leuB strain, the expected band size is 1 kb. Line 3 depicts the SCO obtained with old suicide vector pKH-aac(3’)-DsRed2-KatG-∆MAB_4532c, expected band size 1. kb and 2.5 kb. Line 4 depicts the resulting double mutant after counterselection with isoniazid (M. abscessus ∆ leuB ∆ eis2), expected band size is 4.8 kb. Line 5 depicts the complementation of the leuB, eis2 double deletion with eis2 (new vector pInt), expected band size is 4.8 kb and 10.5 kb. Line 8 depicts the SCO obtained with new pSuc vector, the expected band size is 1 kb and 4 kb, line 9 depicts the double deletion mutant, expected band size is 4.8 kb. Line 10 depicts the complementation with new pInt-amp-eis2 vector, expected band size is 4.8 kb and 10.8 kb.

Figure 4: Whole genome sequence analysis of aac(2’) and eis2 deletion mutants. **a)** genomic region around aac(2’) of the ∆leuB∆aac(2’) mutant. Read coverage for aac(2’) (MAB_4392) is zero (gap 714 bp), corresponding to the deletion of this gene. **b)** genomic region around eis2 of the ∆leuB∆eis2 mutant. Read coverage for eis2 (MAB_4532c) is zero (gap 1232 bp), corresponding to the deletion of this gene.

Figure 5: Phenotypic characterization of aac(2’) and eis2 deletion in leucine auxotrophic M. abscessus strain. **a)** from top left to bottom right: 7H10 agar plate supplemented with L-leucine, all the tested strains can grow (WT, leuB deletion, single cross over, double deletion and complemented double deletion mutant with pInt vector and pInt-aac(2’). Next, 7H10, here only WT, the leuB complemented and SCO with pSuc-Amp-Leu vector could grow. The bottom left depicts 7H10 agar plate supplemented with leucine and apramycin. Here only the SCO with pKH could grow. Bottom right depicts 7H10 with apramycin. No growth is observed for the tested strain. **b)** from top left to bottom right: 7H10 agar plate supplemented with L-leucine, all the tested strains can grow (WT, leuB deletion, single cross over, double deletion and complemented double deletion mutant with pInt vector and pInt-eis2. Next, 7H10, here only WT, the leuB complemented and SCO with complemented and SCO with pSuc-Amp-Leu vector could grow. The bottom left depicts 7H10 agar plate supplemented with leucine and apramycin. Here only the SCO with pkH could grow. Bottom right depicts 7H10 with apramycin. No growth is observed for the tested strain.

Figure 6: Results from drug susceptibility testing and inoculum determination for each replicate for each time point.

Figure 7: Whole genome sequence analysis of gpl genomic region of the ∆leuB∆gpl mutant. Read coverage for 18kb size gpl (MAB_4097c-4099c) is zero, corresponding to the deletion of this gene.

Figure 8: Genealogy of all the generated M. abscessus strains. In light grey color the vectors are depicted.

Table 1: List of strains used in this study.

Table 2: List of plasmids used in this study

Table 3: List of primers used in this study.
